# Rapid Construction of Complex Plant RNA Virus Infectious cDNA Clones for Agroinfection Using a Yeast-*E. coli-Agrobacterium* Shuttle Vector

**DOI:** 10.3390/v9110332

**Published:** 2017-11-07

**Authors:** Kai Sun, Danyang Zhao, Yong Liu, Changjun Huang, Wei Zhang, Zhenghe Li

**Affiliations:** 1 State Key Laboratory of Rice Biology, Institute of Biotechnology, Zhejiang University, Hangzhou 310058, China; sunkai0719@126.com (K.S.); yoyozixiao@163.com (D.Z.); 2 Yunnan Academy of Tobacco Agricultural Sciences, Key Laboratory of Tobacco Biotechnological Breeding, National Tobacco Genetic Engineering Research Center, Kunming 650021, China; haoasliu@163.com (Y.L.); cjhuang@zju.edu.cn (C.H.); 3 Sichuan Plant Protection Station, Chengdu 610041, China; helen68255434@163.com

**Keywords:** infectious cDNA clone, agroinfection, yeast-*E. coli*-*Agrobacterium* shuttle vector, yeast homologous recombination, potyvirus, potato virus Y, rhabdovirus, sonchus yellow net virus

## Abstract

The availability of infectious full-length clone is indispensable for reverse genetics studies of virus biology, pathology and construction of viral vectors. However, for RNA viruses with large genome sizes or those exhibiting inherent cloning difficulties, procedure to generate biologically active complementary DNA (cDNA) clones can be time-consuming or technically challenging. Here we have constructed a yeast-*Escherichia coli*-*Agrobacterium* shuttle vector that enables highly efficient homologous recombination in yeast for assembly of *Agrobacterium* compatible plant virus clones. Using this vector, we show that infectious cDNA clones of a plant negative-stranded RNA virus, sonchus yellow net rhabdovirus, can be rapidly assembled. In addition, one-step assembly of infectious clones of potato virus Y in yeast, either with or without intron, was readily achieved from as many as eight overlapping DNA fragments. More importantly, the recovered yeast plasmids can be transformed directly into *Agrobacterium* for inoculation, thereby obviating the *E. coli* cloning steps and associated toxicity issues. This method is rapid, highly efficient and cost-effective and should be readily applicable to a broad range of plant viruses.

## 1. Introduction

The availability of RNA virus manipulation tools has resulted in enormous advances in our understanding of virus life cycle and pathogenesis. Since Ahlquist and colleagues showed that in vitro RNA transcripts derived from cloned complementary DNAs (cDNAs) of brome mosaic virus are infectious [1], similar approaches were quickly adopted to generate infectious clones for many different plant RNA viruses [2]. These strategies typically involved cloning of viral cDNA sequence downstream of a bacteriophage RNA polymerase promoter (T7, T3 or SP6 etc.) and generation of in vitro run-off RNA transcripts [2]. This method is somewhat cumbersome because care needs to be taken to prevent degradation of RNA transcripts. In addition, initiation of infection with RNA transcripts is less efficient with phloem-limited viruses [3] and not suitable for those viruses with ribonucleoprotein complexes (RNP) as minimal infection unit, for example, negative-stranded RNA viruses (NSVs) [4]. To avoid in vitro transcription steps, Mori et al. [5] demonstrated that recombinant RNA virus can be recovered after mechanical delivery of cloned plasmid DNAs into plant tissues, if viral cDNA was positioned downstream of a cauliflower mosaic virus (CaMV) 35S promoter. This DNA-based inoculation approach was further improved by utilizing the *Agrobacterium*-mediated gene transfer [6,7], in which viral cDNA was inserted within the T-DNA region of an *Agrobacterium* binary shuttle vector is efficiently delivered into plant cells. This technique, known as ‘agroinfection’ or ‘agroinoculation,’ is highly efficient and easy-to-use and quickly became an important alternative for initiating recombinant infections of plant viruses that replicate in hosts susceptible to *Agrobacterium* [8,9].

Although constructing infectious clones for many positive-stranded RNA viruses has become routine, members in the family *Potyviridae* are often recalcitrant to genetic manipulation ([10] and references therein). The difficulties related to the cloning of potyviral genomes include a relatively large genome size (~10 Kb) and the apparently detrimental effects of certain viral sequences on *Escherichia coli* growth. The later issue is probably due to fortuitous expression of toxic viral products directed by prokaryotic promoter-like elements present in potyviral genomes. As a result, spontaneous mutations or deletions of viral sequences occur frequently during propagation of the potyviral cDNA clones in *E. coli* cells [10,11,12,13,14,15,16,17]. Several solutions have been used to overcome the plasmid instability problem, for instance cell-free amplification followed by the biolistic delivery of DNA products into plant cells [18], dividing the viral genome, followed by in vitro ligation prior to inoculation [11], targeted silent mutations to eliminate potential prokaryotic promoter activity [12] and most commonly, the interruption of toxic viral sequences by the insertion of plant intron(s) [10,14,15,16,19,20,21,22]. Although these approaches have been successfully applied to some potyviruses, they are not intuitive and are complex, time-consuming and prone to introducing unwanted mutations. 

In contrast to positive-stranded RNA viruses, whose naked RNAs are infectious after introduction into susceptible cells, obtaining recombinant plant NSVs from cloned cDNA presents additional challenging problems [4]. These difficulties include delivery of nearly exact copies of viral RNA genomes into cells and a requirement for expression of viral core proteins in the same cells for in vivo assembly of biologically active RNPs, which are the minimal infectious units of NSVs [4]. It was not until recently that the first reverse genetics system was developed for a model rhabdovirus, namely sonchus yellow net virus (SYNV) [23]. The procedure involved agroinfiltration of plant leaves for expression of the SYNV antigenomic RNA (agRNA) and the nucleoprotein (N), phosphoprotein (P), large polymerase (L) core proteins. In addition, the co-expression of several viral suppressors of RNA silencing was found to be essential in order to initiating recombinant virus infections [23,24]. The process to engineer infectious SYNV is rather laborious and inefficient, due in part to the SYNV genome size (~14 Kb) and the number of required plasmids (the initial procedure involved as many as 7 plasmid constructs) [23,25]. Therefore, approaches to simplify engineering and to reduce the number of plasmid constructs will facilitate building additional reverse genetics systems for this group of plant viruses.

Conventional methods for infectious clone constructions rely on restriction endonuclease digestion and in vitro ligation, which generally requires tedious sub-cloning steps that can be limited by the availability of common restriction sites shared by the insert and vector. In contrast, homologous recombination (HR)-based cloning in yeast (*Saccharomyces cerevisiae*) is a highly efficient and cost-effective method for seamless assembly of DNA fragments [26,27]. This method, also referred to as gap-repair cloning, requires no enzymes or common restriction sites but takes advantage of yeast endogenous HR machinery for DNA assembly. Circular plasmids can be formed efficiently after co-transformation of yeast cells with DNA fragments and linearized (gapped) vectors, providing that there are short stretches of homologous sequences in both ends of each fragment [28]. Given its simplicity and capacity for complex DNA assembly, yeast homologous recombination has been used in high throughput plasmid construction [29], synthetic genomics and genome engineering [30,31,32]. Assembly of the infectious clones of several plant RNA viruses suitable for biolistic bombardment or agroinfection has also been achieved by HR-based cloning [19,33,34]. 

In this study, we have devised a simplified method for construction of complex plant RNA virus infectious cDNA clones by combining the capacity of yeast HR for large insert cloning and the advantages of agroinfection. For this purpose, we construct a shuttle plasmid able to replicate in *S. cerevisiae*, *E. coli* and *Agrobacterium tumefaciens*. Using this vector, we show that functional full-length SYNV antigenome cDNA clone can be efficiently assembled by yeast HR. In addition, assembly of intron-less and intron-containing infectious clones of potato virus Y (PVY), a type member of the genus *Potyvirus*, was also rapidly achieved in single step. More importantly, the assembled plasmids in yeast can be purified and transformed directly into *A. tumefaciens* for agroinfection, thereby obviating the need for *E. coli* propagation and circumventing concurrent plasmid instability issues. The introns-containing PVY infectious clone permits stable maintenance in *E. coli* and subsequent genetic engineering. The procedure is highly efficient, can be completed within two weeks and requires no expensive reagents.

## 2. Materials and Methods

### 2.1. Virus and Viral RNA

SYNV and a Yunnan isolate of the PVY^N^ strain (Genbank Accession no. MF960848) were propagated in *Nicotiana benthamiana* and *N. tabacum* and the infected plants were maintained in a growth chamber. Total RNAs were extracted from virus-infected plants and used for reverse transcription PCR (RT-PCR) with AMV reverse transcriptase (TaKaRa, Tokyo, Japan) according to the manufacturer’s instructions.

### 2.2. Construction of Yeast-E. Coli-Agrobacterium Shuttle Vector

The yeast-*E. coli*-*Agrobacterium* shuttle vector was constructed on the basis of the binary vector pCB301-HDV backbone [23]. A fragment containing the 2 micro (2μ) yeast replication origin and the *TRP1* autotrophic selection maker gene was amplified from the plasmid pGBK-T7 (Clontech, Shiga, Japan) by PCR using the 2μ ori/F and TRP/R primers (all primer sequences used in this study are provided in Appendix A, Table A1). The purified PCR products were then inserted into an AfeI-linearized pCB301-HDV vector between the Left border and the TrfA gene with an In-Fusion HD PCR Cloning Kit (Clonetech) to produce the shuttle vector pCB301-2μ-HDV. 

To generate pCB301-2μ-eGFP and pCB301-eGFP plasmids, the *eGFP* gene was amplified using the primers eGFP/SalI/F and eGFP/SacI/R. The resulting PCR fragments were digested with SalI and SacI and inserted into pCB301-2μ-HDV and pCB301-HDV, respectively, to replace the hepatitis delta virus (HDV) ribozyme (HDRz) sequence in these vectors.

### 2.3. Assembly of SYNV and PVY cDNA Clones by Yeast Homologous Recombination

To generate pCB301-2μ-SYNV by yeast HR, SYNV antigenomes were divided into four cDNA fragments (A, B, C and D) and amplified with the primer pairs SYNV a/F and SYNV a/R, SYNV b/F and SYNV b/R, SYNV c/F and SYNV c/R and SYNV d/F and SYNV d/R, respectively, using high-fidelity DNA polymerase KOD-Plus-Neo (Toyobo, Osaka, Japan). The plasmid pCB301-2μ-HDV was linearized between the CaMV 35S promoter and the HDRz sequence by PCR with the SYNV backbone/F and SYNV backbone/R primers. To assemble the plasmid by yeast transformation associated recombination, a total of 5 μg DNA containing equal molar ratios of viral cDNA fragments (~0.8 μg each) and 1.8 μg of linearized vector was used to transform yeast competent cells as described previously [35]. Yeast Y2HGold strain (*MATa*, *trp1-901*, *leu2-3*, *112*, *ura3-52*, *his3-200*, *gal4Δ*, *gal80Δ*, *LYS2::GAL1UAS–Gal1TATA–His3*, *GAL2UAS–Gal2TATA–Ade2 URA3::MEL1UAS–Mel1TATA AUR1-C MEL1*) was used for HR. Plasmid assembly by yeast HR was facilitated by the homologous sequences present at both ends of these DNA fragments. The transformed cells were plated on Synthetic Dropout minimal agar media lacking tryptophan and grown at 30 °C for at least 48 h. 

To generate the pCB301-2μ-p19-HCPro-γb plasmid for suppression of host RNA silencing, the expression cassettes of barley stripe mosaic virus (BSMV) γb, tomato bushy stunt virus (TBSV) p19 and tobacco etch virus (TEV) HC-Pro, were each amplified from the pGD-γb, pGD-p19 and pGD-HC-Pro [23], respectively, using the primer pairs 35S-γb/F and NOS-γb/R, 35S-p19/F and NOS-p19/R and 35S-HCPro/F and NOS-HCPro/R. These fragments were assembled with the linearized pCB301-2μ-HDV plasmid by PCR with the primers Backbone/RB/F and Backbone/LB/R. Yeast transformation was performed as described above.

To engineer PVY infectious clones, the total RNA from PVY-infected *N. tabacum* was reverse transcribed with the primer oligo(dT)17 and the PVY full-length cDNA was amplified as three overlapping fragments (A, B and C) with the primer pairs PVY a/F and PVY a/R, PVY b/F and PVY b/R and PVY c/F and PVY c/R, respectively. The plasmid pCB301-2μ-HDV was linearized by PCR with the primers PVY backbone/R and PVY backbone/F to remove the HDRz sequence. Equal molar ratios of linearized yeast vector (2.3 μg) and the three viral cDNA fragments (0.9 μg each) were co-transformed into yeast cells. For the intron-containing PVY clone (pCB-2μ-PVY-intron), the B fragment was further divided into three sub-fragments (B1 to B3) by PCR with the primers PVY b/F and PVY/b1/R, PVY/b2/F and PVY/b2/R and PVY/b3/F and PVY b/R. The intron IV of *ST-LS1* gene was amplified from *Solanum tuberosum* genomic DNA with border modification [36] using the primers intron1/F and intron1/modified/R, followed by a second round of PCR with the primers intron1/F and intron1/R. The intron II of *NiR* gene was amplified directly from *Phaseolus vulgaris* genomic DNA with the intron2/F and intron2/R primers [19]. These two introns were designed to be inserted into the PVY cDNA at nt positions 3517 and 4290, respectively. HR-mediated assembly was performed by co-transformation of yeast cells with approximately equal molar ratios of linearized yeast vector (2.3 μg), five viral cDNA fragments (0.8, 0.2, 0.2, 0.3, 0.8 μg DNA for fragment A, B1-B3 and C, respectively), as well as the two intron fragments (0.1 μg each).

To construct the GFP-tagged PVY clone (The pCB-2μ-PVY-intron-GFP), we utilized two BglII restriction sites located in the PVY *NIb* gene and vector backbone to linearize the plasmid pCB-2μ-PVY-intron. The upstream cDNA region encompassing the BglII site in the 3′ end of the *NIb* gene was amplified with the primers PVY/NIb/BglII/20/F and PVY/NIb/eGFP15/R and the cDNA region encompassing the *NIb* gene proteolytic site (TYEVHHQ) and the downstream BglII site was amplified with the primers PVY/CP/eGFP15/F and PCB/BglII/15/R. The *eGFP* gene was amplified using primers eGFP/F and eGFP/R. All the three PCR products and the linearized vector were designed to share a 15-nt homologous sequence and were assembled in vitro in the presence of an In-Fusion Cloning mixture (Clonetech). 

### 2.4. Yeast Plasmid Purification

Purification of plasmids directly from yeast cells was performed using a modified method [37]. Briefly, Yeast cultures (100 mL) were grown overnight at 30 °C with agitation in yeast extract peptone dextrose (YPD) medium (1% yeast extract, 2% peptone and 2% dextrose). The cells were harvested by centrifugation at 6000× *g* for 15 min at 4 °C. The pellet was re-suspended in 5 mL buffer P1 (Qiagen plasmid midi Kit; QIAGEN, Hilden, Germany). To digest cell walls, 5 mL of lyticase solution was added in this cell suspension and incubated at 37 °C for 1 h and subsequent steps were performed according to the manufacturer’s instructions. 

### 2.5. Agrobacterium Infiltration

Recombinant binary plasmids were mobilized into *A. tumefaciens* strain EHA105 by electroporation. Agrobacteria were grown overnight in Luria-Bertani (LB) media with 10 mM 2-(*N*-morpholino) ethanesulphonic acid (MES), 20 μM acetosyringone and appropriate antibiotics. Before agroinfiltration, suspensions of transformed agrobacteria were adjusted to a 0.7 optical density at 600 nm (OD_600_) in MES buffer (10 mm MgCl_2_, 10 mm MES, pH 5.6, 150 μM acetosyringone) and induced at room temperature for 2 to 4 h. To recover recombinant SYNV, equal volumes of the pGD-NPL, pCB301-2μ-p19-HCPro-γb and pCB301-2μ-SYNV cultures were mixed at 0.7 OD_600_ and infiltrated into *N. benthamiana* plants as previously described [24]. For agroinfection of PVY derivatives, agrobacteria carrying individual recombinant PYV full-length cDNA clones were used to infiltrate *N. tabacum* plants.

### 2.6. Immunoblotting

Total proteins extracted from healthy or infected plant leaves were separated on 12% sodium dodecyl sulfate polyacrylamide gel electrophoresis (SDS-PAGE) gels and transferred to PVDF membranes and analyzed by Western blotting. Polyclonal antibodies for the disrupted SYNV virion [38] or monoclonal antibodies for the PVY CP (prepared in house) were used for virus detections. 

## 3. Results

### 3.1. Construction of a Yeast-E. coli-Agrobacterium Shuttle Vector

**Figure 1:**
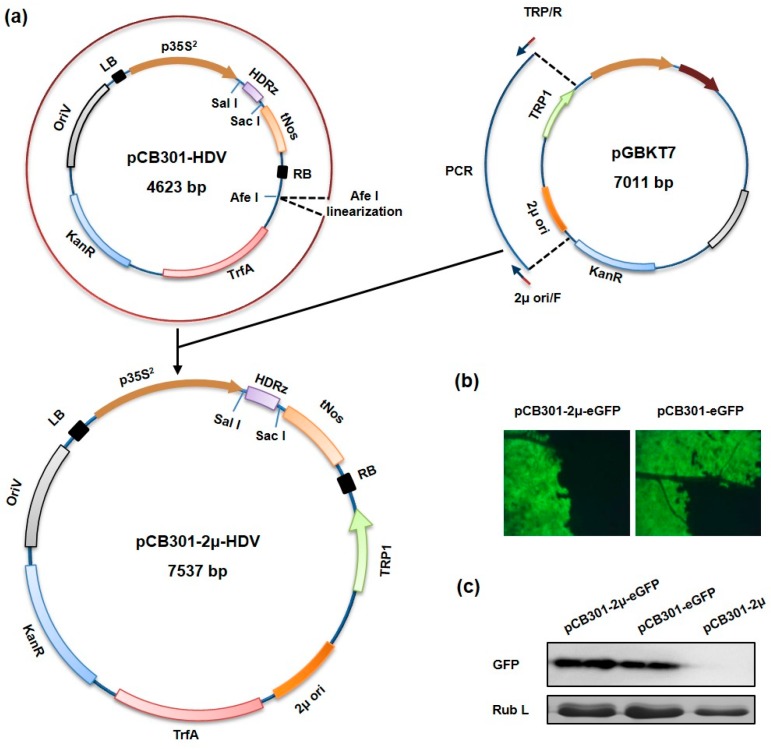
Construction of a yeast-*Escherichia coli*-*Agrobacterium tumefaciens* shuttle vector: (**a**) Schematic diagram illustrating construction of the shuttle plasmid pCB301-2μ-HDV. A fragment containing the yeast 2μ replication origin and *TRP1* selection marker gene was amplified from the yeast plasmid pGBK-T7 with primers TRP/R and 2μ-ori/F and then inserted into the pCB301-HDV *Agrobacterium* binary vector through the AfeI restriction site. p35S^2^, doubled CaMV 35S promoter; (**b**) Transient expression of eGFP in agroinfiltrated leaf patches. *Nicotiana benthamiana* leaves were infiltrated with *Agrobacterium* mixtures carrying the pCB301-2μ-eGFP or pCB301-eGFP plasmid and the TBSV p19 suppressor plasmid. The infiltrated leaves were visualized under a fluorescence microscope at 36 h post infiltration; (**c**) Total proteins extracted from infiltrated leaf patches were analyzed in a Western blot with an eGFP-specific antibody. *TRP1*: tryptophan selection maker gene; OriV: RK2 replication origin; TrfA: replication initiator protein; HDRz: HDV ribozyme; tNos: Nos terminator; 2μ ori: yeast 2μ replication origin; *KanR*: kanamycin resistance gene; LB: left border sequence; RB: right border sequence; eGFP: enhanced green fluorescence protein.

Commonly used *Agrobacterium* binary vectors contain origin(s) of replication capable of replication in both *Agrobacterium* and *E. coli* but lack DNA elements needed to multiply in yeast cells. To construct a shuttle plasmid able to replicate in yeast, *Agrobacterium* and *E. coli*, a small binary plasmid pCB301-HDV, containing the CaMV 35S promoter followed by an HDV antigenomic ribozyme and the Nos terminator within the T-DNA region, was chosen as the vector backbone (Figure 1a). A fragment consisting of the yeast 2μ origin of replication and the *TRP1* autotrophic marker gene was amplified from a yeast shuttle plasmid and inserted into the pCB301-HDV to generate pCB301-2μ-HDV (Figure 1a). The resulting plasmid gained the ability to replicate in yeast cells and allowed auxotrophic yeast strain to grow in tryptophan-minus selection media (data not shown). To show that the novel pCB301-2μ-HDV shuttle plasmid is able to express target gene in plants, an *eGFP* reporter gene was inserted between the 35S promoter and the Nos terminator to generate the pCB301-2μ-eGFP plasmid. Similar construction was made with the original pCB301 vector to produce pCB301-eGFP as a control. Both plasmids were mobilized into *A. tumefaciens* and bacterial suspensions harboring each plasmid were individually infiltrated into *N. benthamiana* leaves to evaluate the eGFP expression. Bright green fluorescence was observed in leaves infiltrated with both the pCB301-2μ-eGFP and pCB301-eGFP at 36 h post infiltration (Figure 1b). Western blot analyses also confirmed a comparable level of eGFP proteins expression (Figure 1c), suggesting that the insertion of the yeast replication signal and selection marker gene does not compromise level of transient expression. 

### 3.2. Assembly of SYNV cDNA Clones by Yeast Homologous Recombination

**Figure 2:**
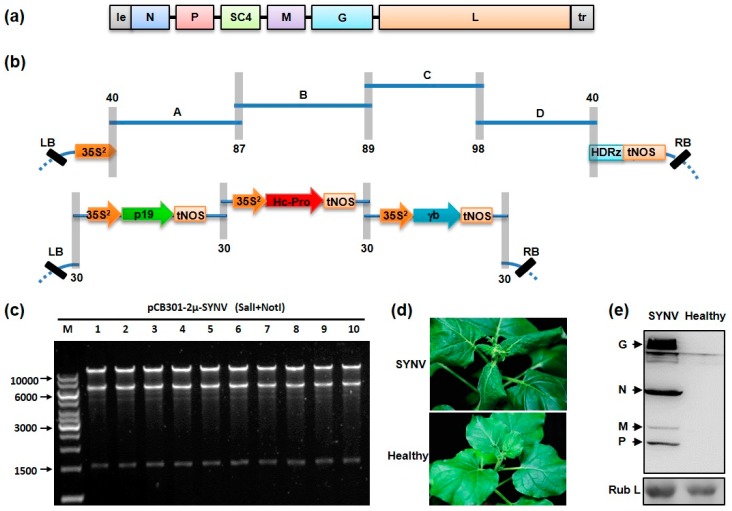
Construction of cDNA clones by homologous recombination (HR) in yeast for recovery of recombinant sonchus yellow net virus (SYNV): (**a**) Diagram depicting the SYNV antigenomic RNA. Six viral genes (*N*, *P*, *sc4*, *M*, *G* and *L*) are flanked by the 5′ leader (le) and 3′ trailer (tr) sequences; (**b**) Schematic representation of the yeast HR-based cloning strategy. In the upper panel, four overlapping SYNV cDNA fragments (A, 3584 bps; B, 3637 bps; C, 3370 bps; D, 3439 bps) and the linearized vector pCB301-HDV-2μ are shown. After assembly, viral cDNA is positioned precisely between the 35S promoter (35S^2^) and HDV ribozyme (HDRz). The lower panel shows the assembly scheme for the tandem arrayed expression cassettes of the tomato bushy stunt virus p19, tobacco etch virus HC-Pro and barley stripe mosaic virus γb viral suppressors of RNA silencing. The overlapping regions are depicted by gray shadow boxes, with the number of overlapping nucleotides indicated. Note that all of the fragments in the figure are not drawn exactly to scale; (**c**) SalI and NotI double digestion of ten recombinant plasmids. The positions of the 10,000, 6000, 3000 and 1500 bps size marker (M) are indicated to the left of the panel; (**d**) Symptoms of *N. benthamiana* plant infected by recombinant SYNV at 35 days post infiltration. Healthy: an uninfected plant; (**e**) Total proteins extracted from systemically infected leaf tissues were analyzed by Western blotting using an antibody against SYNV virions. The Coomassie blue-stained Rubisco larger submit (Rub L) is shown as loading control.

To verify the capacity of yeast HR-based cloning for assembly cDNA clones of complex plant RNA viruses, we first attempted to clone the SYNV full-length agRNA, which is ~14 Kb in length. SYNV antigenome was amplified by RT-PCR from infected *N. benthamiana* to produce four overlapping cDNA fragments (A, B, C and D) and the vector DNA was linearized by PCR. These fragments contain at least 30-bp terminal homologous sequence to facilitate assembly of the circularized plasmid as outlined in Figure 2b. Approximately equal molar ratios of linearized vector and the four viral DNA fragments were transformed into yeast cells. After incubation in selective media, over 100 yeast colonies were recovered. Recombinant plasmids from ten randomly selected yeast colonies were isolated and subjected to further analyses. After digestion with SalI and NotI enzymes, all ten plasmids generated expected restriction patterns (Figure 2c). Junction sequences between each overlapping fragments were amplified from two representative plasmids and correct assemblies were confirmed by directly Sanger sequencing (data not shown). In these assembled pCB301-2μ-SYNV plasmids, viral cDNA was positioned under the control of the 35S promoter to produce antigenomic viral transcript with authentic 5′ end and HDV ribozyme cleavage generates precise viral 3′ end (Figure 2b).

Our previous studies have shown that recovery of recombinant SYNV requires simultaneous expression of the viral agRNA and nucleocapsid core proteins in single cells. In addition, suppression of host RNA silencing activities by co-expression of three suppressors of RNA silencing, namely the BSMV γb, TBSV p19 and TEV HC-Pro, is necessary for recovery [23]. To reduce the number of plasmids and simplify the infiltration process, we combined the three suppressors in a single multi-protein expression vector. For this purpose, each of the suppressor expression cassettes containing the 35S promoter, the coding region and the Nos terminator sequence were amplified from respective binary vectors. The pCB301-2μ-HDV vector backbone was also linearized by PCR (Figure 2b). These overlapping DNA fragments were circularized after co-transformation into yeast cells and the assembled recombinant plasmid pCB301-2μ-p19-HCPro-γb was immobilized into *A. tumefaciens*.

To test the efficiency of recombinant SYNV recovery, we co-infiltrated *N. benthamiana* leaves with mixtures of three agrobacterial strains harboring the pCB301-2μ-SYNV and pCB301-2μ-p19-HCPro-γb plasmids, along with a strain with the pGD-NPL supporting plasmid that was constructed previously to express the N, P and L core protein [23]. At about 25 days post infiltration (dpi), SYNV symptoms began to appear on newly developed, un-inoculated upper leaves and the leaf symptoms coincided with typical dwarfing and leaf shrinking (Figure 2d). Ultimately, 9 of 40 infiltrated plants developed systemic infections. Western blot analyses with a polyclonal antibody against SYNV virions revealed accumulation of viral structural proteins in tissue extracts of systemically infected leaves (Figure 2e). The 22.5% infection rate observed in this study is higher than that in previous reports in which 5–7 plasmids were infiltrated [23,25]. In addition, the assembly procedure is simple, efficient and takes less than two weeks to complete.

### 3.3. One-Step Construction of Intron-Less and Intron-Containing PVY Infectious cDNA Clones

**Figure 3:**
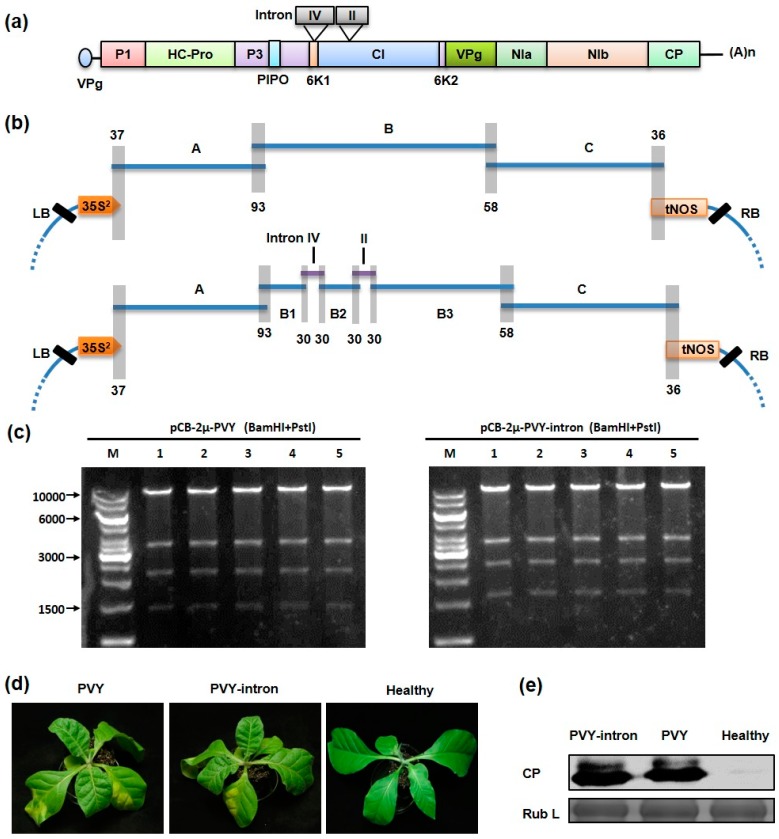
One-step assembly of intron-less and intron-containing potato virus Y (PVY) infectious cDNA clones by homologous recombination in yeast: (**a**) PVY genome structure. The 5’ viral protein genome-linked (vpg) and the 3′ polyadenylated tail [(A)n] are depicted. The positions of two inserted introns are indicated on the top of the genome; (**b**) Schematic representation of the cloning strategies for yeast homologous recombination-mediated assembly of the PVY intron-less (upper panel) and intron-containing (lower panel) full-length clones. The intron-less PVY clone was assembled by co-transformation of yeast cells with the three overlapping PVY cDNA fragments (A, 3311 bps; B, 3359 bps; C, 3259 bps) and the appropriately linearized vector pCB301-HDV-2μ. For the intron-containing clone, the B fragment is divided into three fragments (B1, B2 and B3) by insertion of the two inserted intron (IV and II). The overlapping regions are depicted by gray boxes, with the number of overlapping nucleotides indicated. Note that all of the fragments in the figure are not drawn exactly to scale; (**c**) Recombinant plasmids were digested with BamHI and PstI and the products were separated on 0.8% agarose gels. The positions of the 10,000, 6000, 3000 and 1500 bp size markers (M) are indicated to the left of the panel; (**d**) *Nicotiana tabacum* plants systemically infected with the intron-less or intron-containing PVY infectious clone were photographed at 15 days post infiltration. Healthy: an uninfected plant; (**e**) Total proteins extracted from PVY infected plants were analyzed by Western blotting using an antibody against the PVY CP protein. The Coomassie blue-stained Rubisco large subunit (Rub L) is used as a loading control.

The PVY genome has been proved to be difficult to clone due to the presence of toxic sequences in *E. coli*. To avoid this issue, we set out to construct intron-less and intron-containing PVY infectious cDNA clones using the yeast HR-based cloning. For the construction of the intron-less clone, we divided the PYV genome into three partially overlapping cDNA fragments (A to C) (Figure 3a). These viral cDNA fragments were amplified by RT-PCR using total RNA extracted from PVY infected *N. tabacum* plants, followed by yeast transformation and HR assembly with the linearized pCB301-2μ-HDV vector to produce the pCB-2μ-PVY (Figure 3b). To construct the intron-containing PVY clone (pCB-2μ-PVY-intron), the B fragment was further divided into three sub-fragments (B1 to B3) by incorporation of two plant introns, i.e., the intron IV of *ST-LS1* gene from *S. tuberosum* and intron II of *NiR* gene from *P. vulgaris* [19], which were designed to interrupt the 6K1 and CI coding regions, respectively (Figure 3a). The five viral cDNA fragments (A, B1–B3 and C), as well as the two intron DNAs, were co-transformed into yeast cells along with the linearized yeast vector. In both cases, dozens of yeast colonies were obtained on tryptophan-minus media. Five yeast colonies from each transformation were selected and inoculated into yeast liquid media under selective pressure and recombinant plasmids were isolated from the yeast cultures. Upon digestion with BamHI and PstI, all these plasmids displayed the expected restriction patterns (Figure 3c). 

The verified pCB-2μ-PVY and pCB-2μ-PVY-intron recombinant plasmids were introduced directly into *A. tumefaciens* separately to evaluate infectivity by agroinfection. At 6 dpi, three out of ten *N. tabacum* plants inoculated with the pCB-2μ-PVY-intron exhibited leaf mottling symptoms in the newly emerged leaves and by 8 dpi, all inoculated plants (10/10) developed systemic infections. Symptom development in tobacco plants infiltrated with the pCB-2μ-PVY clone were similar to those with the intron-containing clone but disease onset was generally delayed by one day: systemic symptoms was observed at 7 dpi in two out of ten inoculated plants and it is was not until 9 dpi that 100% of inoculated plants (10/10) became infected. Nevertheless, all the infected plants showed symptoms typical of PVY infection (Figure 3d) and similar level of coat protein (CP) were detected in systemically infected tissues by Western blotting (Figure 3e). 

### 3.4. Construction of an eGFP-Tagged PVY Infectious Clones

**Figure 4:**
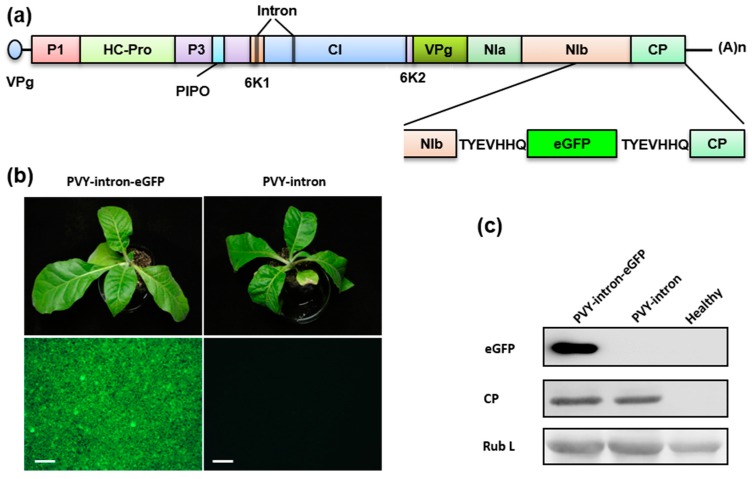
Construction of an eGFP-tagged PVY intron-containing infectious cDNA clone: (**a**) Schematic representation of the *eGFP* insertion; (**b**) Symptoms and eGFP expression of PVY-intron-GFP and PVY-intron infected *N. tabacum* plants. The upper panels show symptomatic plants photographed at 15 days post infiltration and the lower panels show eGFP expression in systemically infected leaf tissues observed by fluorescence microscope; (**c**) Total proteins extracted from systemically infected plants or un-infiltrated control plants were analyzed by Western blotting using an antibody against the PVY coat protein (CP). The Coomassie blue-stained Rubisco large subunit (Rub L) is showed as a loading control.

To test whether the intron-containing PVY clone is stable in E. coli, the pCB-2μ-PVY-intron clone was propagated in *E. coli* and the *eGFP* gene was inserted into the recovered plasmid between the PVY nuclear inclusion b (NIb) and the CP coding regions. To facilitate post-translational release of eGFP from viral polyprotein precursor, the inserted *eGFP* sequence was flanked by two identical proteolytic sites (TYEVHHQ) (Figure 4a). We did not observe any adverse effects of the PVY-intron derived clones on bacteria growth and the recombinant plasmids were easily recovered. The resulting PVY-intron and PVY-intron-GFP clones were transformed into *A. tumefaciens* and their infectivities were evaluated separately after agroinoculating *N. tabacum* plants. Both clones were highly infectious on 8 of 8 inoculated tobacco plants and induced mottling and mosaic symptoms (Figure 4b). The infected plants had a similar level of PVY CP accumulation in systemically infected leaf tissues (Figure 4c). Expression of eGFP in the PVY-intron-GFP infected tobacco plants was readily detectable by fluorescence microscopy (Figure 4b) and Western blotting (Figure 4C). In summary, our data show that the PVY-intron clone is amenable to genetic manipulation in *E. coli* and that the PVY infectious clone can be engineered for stable expression of a reporter gene. The eGFP-tagged clone should be a useful tool for non-invasive tracking of PVY infection and further investigations of virus-host interactions.

## 4. Discussion

To generate biologically active RNA virus infectious clones, it is often necessary to position viral cDNAs precisely between an appropriate promoter and a 3′ processing ribozyme for the synthesis of viral RNAs with authentic 5′ and/or 3′ termini [39,40]. Such constructions can be achieved by classical restriction digestion-based cloning methods but usually require sophisticated designing and a number of sub-cloning steps. Recently, several ligation-independent cloning methods—for instance the In-Fusion cloning and Gibson Assembly—have been used to construct plant virus infectious clones [10,41,42,43]. Although these in vitro seamless assembly methods permit simple, straightforward and efficient approaches to vector construction, the commercial reagents used in these methods are quite expensive, especially for high-throughput construction experiments. Yeast HR-based cloning provides alternative strategy for scarless assembly of multiple DNA fragments. Only 20 to 30 bp homologous ends flanking the fragments are sufficient to guide yeast endogenous machinery to assemble recombinant constructs. The efficiency and capacity of this method for complex DNA construction is best manifested by a study showing that a complete mycoplasma genome of 1.08 mega base pairs (Mb) was successfully obtained by HR in yeast [32]. The yeast HR-based method has also been used to construct infectious cDNA clones of plant and animal RNA viruses with relatively small genomes [19,33,34,44]. Recently, a synthetic genome of *Autographa Californica* nucleopolyhedrovirus, an insect baculovirus with large circular double-strand DNA genome of 145 Kb, has been assembled into an infectious DNA clone by using yeast HR [45], further demonstrating the usefulness of this method for complex viral clone construction.

In this study, we have harnessed the power of yeast HR for assembly of plant viral cDNAs and to enable direct transfer of cloned viral cDNAs into *Agrobacterium* for infection. We constructed a hybrid shuttle vector capable of replicating in *S. cerevisiae*, *E. coli* and *A. tumefaciens*. As a proof of concept, we show that assembly of infectious cDNA clones of two complex RNA viruses, namely, SYNV and PVY, can be achieved in less than 2 weeks. As many as eight overlapping DNA fragments can be efficiently joined together to form corrected assembled circular plasmids after one-step yeast transformation. Our method is simple and efficient, requires no extraordinary skill, expensive supplies or equipment. In addition, the method helps overcome plasmid toxicity issue during *E. coli* propagation because plasmids recovered from yeast can be used directly for *Agrobacterium* transformation. In theory, any conventional *Agrobacterium* binary vector can be easily converted to yeast shuttle vector by insertion of an autotrophic selection marker and a yeast origin of replication. We anticipate that this method can be readily adapted to reverse genetics analysis of a broad range of plant viruses, providing that an agroinfiltration host is available and that toxicity issue do not affect *Agrobacterium*.

The ability of yeast HR to assemble large inserts from multiple fragments will be particularly useful for constructing infectious clones for plant NSVs, whose genomes are relatively large (11–26 Kb) and consist of one to six segments [46]. Although amplification of cDNAs up to 10 Kb by RT-PCR is possible nowadays with robust commercial reagents, construction of such cDNAs in binary vectors by conventional cloning methods is still tedious. Additional technical difficulties associated with NSVs include the requirement for a large number of plasmids to be delivered into host cells to provide viral genomic RNA segment(s), supporting viral core proteins [4,39]. In the case of plant NSVs, the situation is further complicated by the fact that co-expression of three RNA silencing suppressors is usually required for successful recovery [23]. Because multiple plasmids need to be present in the same cells to reconstitute biologically active RNPs, the generation of a recombinant virus is probably a rare event, which may account for the observed low recovery efficiency [23,39]. We previously reported that combined expression of the SYNV N, P and L proteins in a single construct to reduce the number of plasmids from seven to five, resulted in approximately 2-fold increased infection rates in agroinfiltrated plants [23]. Similarly, recent studies with an avian mononegavirus, Newcastle disease virus, have shown that this strategy not only permits accelerated and increased production of recombinant viruses but also enables recovery of attenuated viral strains [47,48], highlighting the efficiency of using lower numbers of plasmids. In this study, a single supporting plasmid designed to express the three viral suppressors was constructed by using the yeast HR-mediated cloning. Compared to the five-plasmid system [23], the frequency of recombinant virus recovery with the three-plasmid system was further increased by around 2-fold. In the future, it will be worthwhile to test whether combining all of the viral components in a single construct could further increase the infection efficiency, as a similar strategy has proven to be effective for rescuing the eight-segmented influenza virus [49,50]. The simple and effective yeast HR-based cloning system would be particularly helpful for such constructions.

The use of yeast and *Agrobacterium* hybrid shuttle vectors offers added advantage for cloning those viral genomes that are apparently toxic to *E. coli*. PVY—as well as many other members in the family *Potyviridae*—is notoriously difficult to clone mainly due to toxicity-associated issues [15,16,42]. Although intron insertion has been widely used to stabilize potyviral clones in *E. coli*, the number of introns and the location of insertions has to be determined empirically for each potyviruses [14,20,21,22]. As a result, only limited success has attained for this group of viruses and a stable infectious clone is unavailable for a large number of potyviral members that collectively account for many economically important crop diseases. Here we show that an intron-less PVY cDNA clone can be assembled rapidly by yeast HR and that the clone is highly infectious after direct transformation into *A. tumefaciens* for agroinfection. A recent study also reported generation of intron-less potyviral infectious clones compatible with agroinfection [42]. In this study, Gibson Assembly was used to join the viral cDNA in an *Agrobacterium* binary vector and the in vitro assembled DNA products were transformed directly into *A. tumefaciens*, thereby bypassing the plasmid instability issue in *E. coli*. Thus, these “*E. coli*-free” and intron-less cloning approaches provide new strategies for rapid generation of potyviral infectious clones and circumvent complicate plasmid instability issues. In addition, efficient infection can be achieved using the cost-effective and easy-to-use agroinfection method. The availability of such clones for those less-well studied potyviral members will be very useful to fulfill Koch’s postulates, to determine virus pathogenicity and to screen resistant crop varieties. However, it is desirable to have a stable infectious clone in *E. coli* when repeated genome manipulation attempts are needed. As documented frequently in previous studies [15,16,42], intron-less PVY full-length clones are instable in *E. coli* (data not shown). Here we show that an intron-containing PVY clone can also be efficiently assembled in yeast from as many as eight overlapping DNA fragments. As expected, insertion of introns stabilized the PVY clone in *E. coli* and rendered it amenable for genetic manipulation such as eGFP tagging.

## Appendix A

**Table A1:** List of primers used in the study.

Primer	Sequence ^1^
2μ ori/F	agatctcagtaaagcGAAAAGTGCCACCTGAACGA
TRP/R	gggggttcagccagcAAGGATCTGGCGTAATAGCG
eGFP/SalI/F	GC*GTCGAC*ATGGTGAGCAAGGGCGAGGA
eGFP/SacI/R	GC*GAGCTC*TTACTTGTACAGCTCGTCCAT
Backbone/LB/R	AAAACCACCCCAGTACATTAAAAAC
Backbone/RB/F	GAATTCAGATTGTCGTTTCCCG
35S-p19/F	CGGACGTTTTTAATGTACTGGGGTGGTTTTACGCGTCATGGTGGAGCACG
NOS-p19/R	GTCTAGAGAATCTCCTGCAATCATTCCGGAAACACTGATAGTTTAATTCCCGAT
35S-HCPro/F	GGAGGGACTGTATTCATGTTAAATGATAAGACGCGTCATGGTGGAGCACG
NOS-HCPro/R	ACTGAAGGCGGGAAACGACAATCTGAATTCAACACTGATAGTTTAATTCCCGAT
35S-γb/F	TCCGGAATGATTGCAGGAGATTCTCTAGACACGCGTCATGGTGGAGCACG
NOS-γb/R	CTTATCATTTAACATGAATACAGTCCCTCCAACACTGATAGTTTAATTCCCGAT
oligo(dT)17	GACTCGAGTCGACATCGATTTTTTTTTTTTTTTTT
PVY backbone/R	GTATTGAGTTGTTTTAATTTCCTCTCCAAATGAAATGAACTTCCT
PVY backbone/F	GATGTCGACTCGAGTCGAATTTCCCCGATCGTTCAAACATTTG
PVY a/F	CATTTCATTTGGAGAGGAAATTAAAACAACTCAATACAACATAAG
PVY a/R	TATTGCCTGACACACTGCCA
PVY b/F	ATCTTTAGGCGTTTGCCAAC
PVY b/R	ACAGGGAAATCCTTTGGCATC
PVY c/F	TTTTACCGATCAAAGGCAGAGAC
PVY c/R	TTGAACGATCGGGGAAATTCGACTCGAGTCGACATCGATT
PVY/b2/F	TTTGTTGATATGCAGGTGTTAAAAATTTGGAACAAGTGGT
PVY/b1/R	GAAGCAGAAACTTACCTGGTGTGGAACGCTGGTGT
PVY/b2/R	TAAGTGCATACTTACCTCTCTTCCCACTGGAGTAGCT
PVY/b3/F	TTTGAAATTATGCAGGTTGAATTTACAACACAGCAACCA
Intron1/modified/R	CTGCATATCAACAAATTTTGGTCATATATTAGAAAAGTTATAAATTAAAAT
Intron1/F	AGCGTTCCACACCAGGTAAGTTTCTGCTTCTACCTTTGAT
Intron1/R	CCAAATTTTTAACACCTGCATATCAACAAATTTTGGTCAT
Intron2/F	CCAGTGGGAAGAGAGGTAAGTATGCACTTAAAGAGTATGTGT
Intron2/R	TGTTGTAAATTCAACCTGCATAATTTCAAAGATTGAACCTA
SYNV backbone/F	TGTTCTGAGCTTTTGTCTCTGGGTCGGCATGGCATCTCCA
SYNV backbone/R	TTTTCTGAGTTTCTGTCTCTCCTCTCCAAATGAAATGAACTTC
SYNV a/F	GTTCATTTCATTTGGAGAGGAGAGACAGAAACTCAGAAAATACAAT
SYNV a/R	CACATCCTCAAGAAACATGCT
SYNV b/F	CCATTTGCCATGATCAGACG
SYNV b/R	GATGGGTCGTTTGATCGATG
SYNV c/F	GCTTCTGAACGACATCTGAATC
SYNV c/R	CCGATAGATCTCGCAAATATTGAT
SYNV d/F	CAAAGCAGAGGCCGTAATGAG
SYNV d/R	TGGAGATGCCATGCCGACCCAGAGACAAAAGCTCAGAACAAT
PVY/NIb/BglII/20/F	tcacaacatttctc *agatcT*TGGTTTG
PVY/NIb/eGFP15/R	gcccttgctcaccatTGCTTGATGGTGTACTTCATAAGTATCGC
PVY/CP/eGFP15/F	gacgagctgtacaagACTTATGAAGTACACCATCAAGCAAAT
PCB/BglII/15/R	cgctttactg *agatcT*CCTGTG
eGFP/F	ATGGTGAGCAAGGGCGAGGA
eGFP/R	TTACTTGTACAGCTCGTCCATGCCGA

^1^ The sequence shown in lower letters is homologous to other primer to facilitate In-Fusion cloning; the overlapped restriction site is in italics. Underlined sequence is homologous to other primer to facilitate homologous recombination in yeast.
